# Retraction Note: Prenatal Exposure to COVID-19 mRNA Vaccine BNT162b2 Induces Autism-Like Behaviors in Male Neonatal Rats: Insights into WNT and BDNF Signaling Perturbations

**DOI:** 10.1007/s11064-025-04492-x

**Published:** 2025-07-19

**Authors:** Mumin Alper Erdogan, Orkun Gurbuz, Mehmet Fatih Bozkurt, Oytun Erbas

**Affiliations:** 1 Faculty of Medicine, Department of Physiology, Izmir Katip Celebi University, Izmir, Turkey; 2 Department of Radiotherapy Programme, Istinye University, Istanbul, Turkey; 3 Faculty of Veterinary Medicine, Department of Pathology, Afyon Kocatepe University, Afyon, Turkey; 4 Faculty of Medicine, Department of Physiology, Demiroğlu Bilim University, Istanbul, Turkey

**Retraction Note: Neurochemical Research (2024) 49:1034–1048**

10.1007/s11064-023-04089-2

The Editor-in-Chief has retracted this article. After publication, several concerns were raised regarding the methodology described in this article. The authors have provided raw data for validation. However, a post-publication review found inconsistencies in the number of subjects reported in the Methods and raw data. The Editor-in-Chief therefore no longer has confidence in the presented data.

None of the authors have responded to correspondence regarding this retraction.

